# Oxidation of Ca-α-SiAlON Powders Prepared by Combustion Synthesis

**DOI:** 10.3390/ma8115394

**Published:** 2015-11-11

**Authors:** Jinfu Li, Zhongmin Li, Enhui Wang, Zhanjun Wang, Xiaowei Yin, Zuotai Zhang

**Affiliations:** 1 School of Materials Science and Engineering, Northwestern Polytechnical University, Xi’an 710072, China; leesanqiang@yahoo.com (J.L.); yinxw@nwpu.edu.cn (X.Y.); 2 Department of Energy and Resources Engineering, College of Engineering, Peking University, Beijing 100871, China; pkulzm@163.com; 3 School of Metallurgical and Ecological Engineering, University of Science and Technology Beijing, Beijing 100083, China; wangeh1990@163.com (E.W.); wangzhanjun1990@163.com (Z.W.)

**Keywords:** combustion synthesis, oxidation, Ca-α-SiAlON, additives

## Abstract

The oxidation of Ca-α-SiAlON synthesized by the combustion synthesis (CS) method with different additives was investigated in air atmosphere using thermogravimetric (TG) analysis in a temperature range from 1453 K to 1653 K. The experimental results indicated that oxidation was controlled by mixed chemical and diffusion steps. The oxidation products by XRD analysis were composed of SiO_2_ and CaAl_2_Si_2_O_8_ at low oxidation temperature, whereas the SiO_2_-Al_2_O_3_-CaO ternary glassy phase was formed at elevated temperature. The deviation of oxidation resistance from each sample may be due to the morphological difference brought about by different additive additions. This study reveals the effects of additives on the oxidation resistance of synthesized Ca-α-SiAlON powders.

## 1. Introduction

It is well known that the SiAlON ceramics are candidate materials for high temperature structural applications in cutting tools, abrasive materials, and the refractory industry because of their outstanding combination of mechanical and high temperature properties [1,2,3,4,5]. Generally speaking, SiAlON materials include a large group of phases in Si-Al-O-N and M-Si-Al-O-N (M represents a metal element) systems, where α-SiAlON and β-SiAlON are two well-studied phases with particular important properties [6,7,8,9]. Currently, numerous advanced technologies have been exploited and developed to synthesize SiAlON ceramics, such as the carbothermal reduction nitridation method [10], solid state reaction using Al_2_O_3_, AlN and Si_3_N_4_ as raw materials in a nitrogen atmosphere [11], combustion synthesis (CS) [12], metal reduction nitridation [13], *etc*. Among these methods, combustion synthesis (CS) (*i.e.*, self-propagating high temperature synthesis) has attracted great attention on account of its capacity for energy saving, short reaction time, and its ability to form high purity products [12]. In view of these developed methods, the application of SiAlON has achieved wide use in industry, especially in high temperature industry, because of their excellent performance as engineering ceramics, cutting tools, and refractory materials.

For long-term operation under high temperature conditions, oxidation resistance is crucial for the practical uses of SiAlON ceramics, a subject that therefore attracted great attention during past decades. Chou *et al.* [14,15,16,17,18] have developed an oxidation model based on the explicit derivation of gas-solid chemical reaction and successfully applied the model in describing the oxidation process of SiAlON materials. Akiyama *et al.* [19] studied the oxidation behaviors of β-SiAlON synthesized by a combination of combustion synthesis and spark plasma sintering. Shimada *et al.* [20,21] investigated the early-stage oxidation behavior of carbothermally synthesized β-SiAlON powders and found that the oxidized products were composed of silica at the early stage and were followed by the formation the amorphous aluminosilicate. In recent years, the oxidation behaviors of transparent SiAlON ceramics and novel SiAlON powder have also been reported [22,23], which extended the application field of SiAlON from a structural material to a functional one. MacKenzie *et al.* [23] investigated the kinetics and mechanism of the thermal oxidation of SiAlON ceramic powders prepared by silicothermal synthesis. These studies are beneficial for the further application of SiAlON materials in industry. However, to the best knowledge of the present authors, no systematic investigations on the oxidation behaviors of Ca-α-SiAlON materials prepared by combustion synthesis method have been carried out so far, thus providing motivation for the present study. Furthermore, it is well known that additives are often used to promote the reaction process, while the additives also have a significant effect on morphological properties and further affect the oxidation behaviors of SiAlON powders [12]. How the additives affect oxidation behavior is also important for the further utilization of Ca-α-SiAlON powders. Therefore, the purpose of this paper was to investigate the oxidation performance of Ca-α-SiAlON powders synthesized by combustion synthesis, and the effects of the addition of additives on the oxidation behavior of materials. The present study aims to provide an understanding of the design of Ca-α-SiAlON for engineering applications at high temperatures.

## 2. Results and Discussion

### 2.1. Characterization of Ca-α-SiAlON Powders

**Figure 1:**
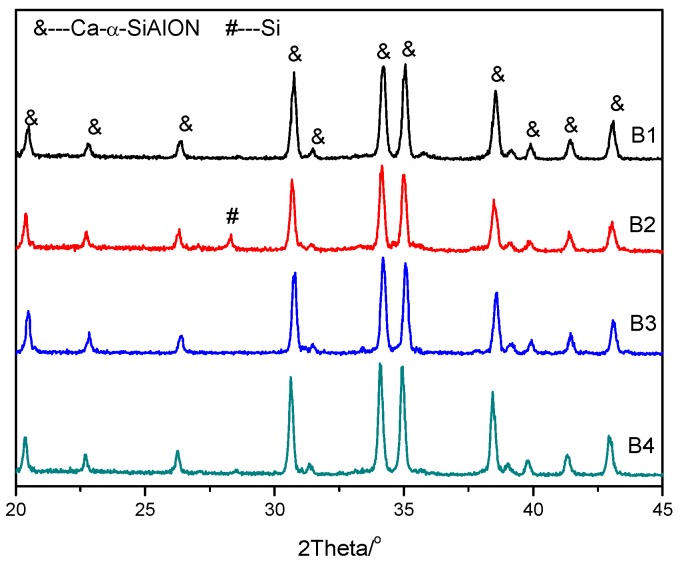
XRD patterns of combustion synthesized Ca-α-SiAlON with different additives.

**Figure 2:**
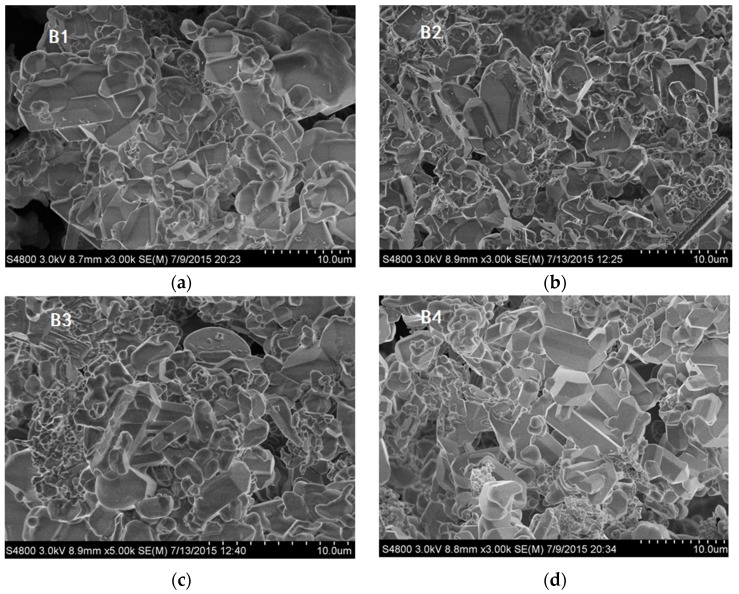
SEM images of combustion synthesized Ca-α-SiAlON with different additives: (**a**) sample B1; (**b**) sample B2; (**c**) sample B3 and (**d**) sample B4.

**Figure 3:**
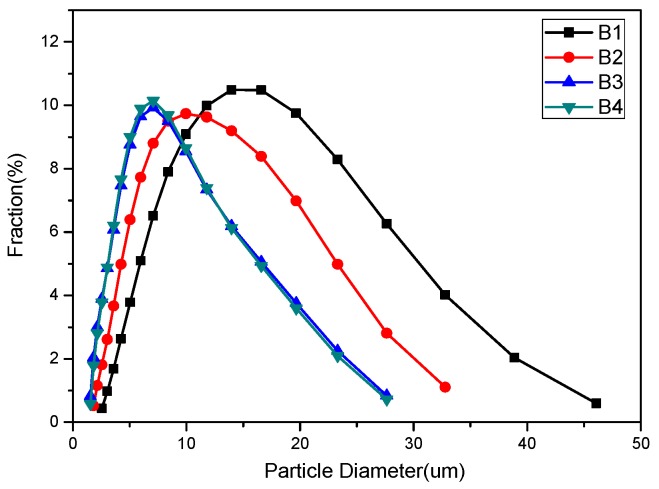
Distribution of particle diameter of combustion synthesized Ca-α-SiAlON with different additives.

The XRD patterns of different samples are shown in Figure 1, and it can be seen that pure phase Ca-α-SiAlON was produced, except for sample B2, which contained a small amount of residual Si, estimated to be around 2%. This is consistent with previous results showing that the addition of a small amount of Ca^2+^ results in higher stability in α-Sialon [12]. Figure 2 shows SEM images of as-synthesized Ca-α-SiAlON powders, consisting of isotropic grains. It can be seen that grain size and morphology in different samples were not at all similar. Fine isotropic grains with a small amount of hexagonal column-shaped crystals were obtained in samples B3 and B4, while much larger isotropic grains were obtained in sample B1. The existence of hexagonal column-shaped crystals is due to the presence of Ca^2+^ as the modifying cation, which has been proven to promote the formation of hexagonal column-shaped microcrystals [12]. This has been shown by the distribution of particles determined by laser interferometer (SEISHIN LMS-30). Figure 3 shows the density function of particle size for different samples; it can be seen that the particle size of samples B3 and B4 are identical from 2 μm to 28 μm, while sample B1 shows a much larger particle size distribution from 3 μm to 46 μm.

**Figure 4:**
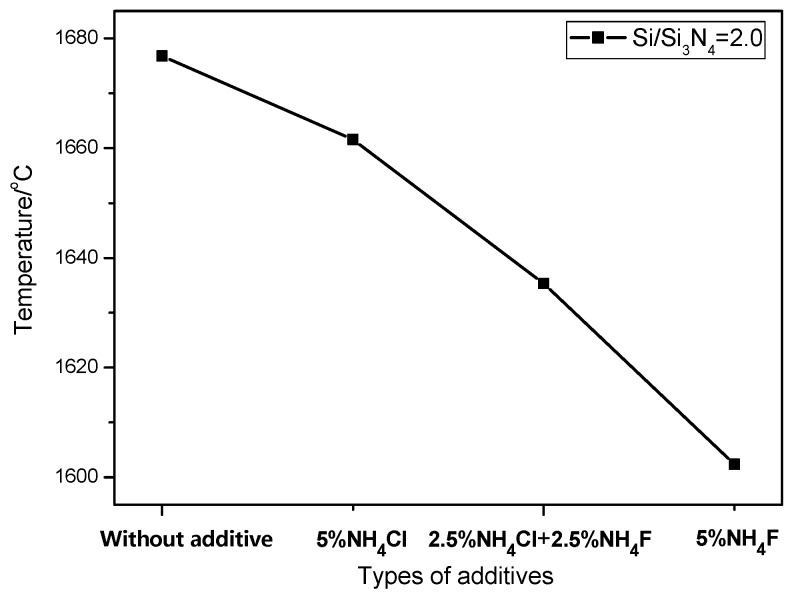
Temperature of combustion synthesis with different additives.

During the combustion synthesis process, the reaction temperature was drastically increased to above 1600 °C (Figure 4), which is beneficial as it accelerates reaction rate and avoids the formation of an intermediate phase. In order to control the synthesis process, ammonium salts, such as NH_4_F or NH_4_Cl, were usually added in order to control material morphology in different aspects. It is well known that the decomposition of NH_4_F or NH_4_Cl is an endothermic process, resulting in a decreased combustion temperature. It is also noted that the enthalpy of NH_4_F decomposition is higher than that of NH_4_Cl, resulting in a lower combustion temperature than that of NH_4_Cl (*i.e.*, the combustion temperature for sample B4 was lower than that for sample B2). Due to the low combustion temperature for sample B2, the small amount of Si could not be completely nitridated and were therefore detected in the XRD pattern (Figure 1). Furthermore, NH_4_F or NH_4_Cl also acted as a catalyst [24]; the decomposed nitrogen promoted the nitridation reaction of Si and led to enhanced Si_3_N_4_ formation.

It is also noteworthy that nucleation is closely related to temperature. With the lowest combustion temperature in sample B2, more crystal nuclei of Ca-α-SiAlON could precipitate from the liquid phase. Meanwhile, due to the space restrictions for the growth of Ca-α-SiAlON, the morphology of the crystal changed into grains and the Ca-α-SiAlON particle size decreased. Compared to Sample B2, higher combustion temperature caused a longer reaction time for Sample B3, which benefited the growth of the crystal in the [0001] direction and promoted the formation of hexagonal column-shaped α-SiAlON. With the addition of mixed additives, enhanced formation of Si_3_N_4_ provided more nucleation sites and caused the enhancement of granular particles. On the other hand, hexagonal column-shaped crystals were formed due to the addition of NH_4_Cl.

### 2.2. Oxidation Behavior

**Figure 5:**
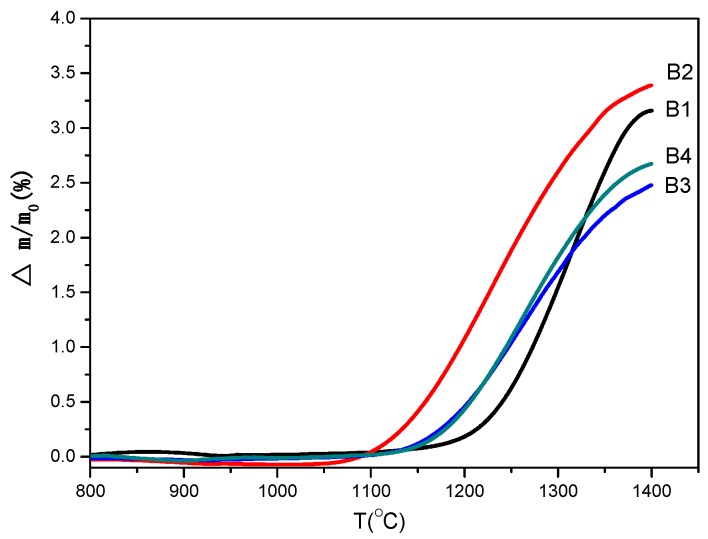
Non-isothermal oxidation curves for different samples.

In order to determine oxidation characteristics and choose the oxidation temperature range, the non-isothermal experiments were conducted under air atmosphere, and the results are shown in Figure 5. It can be seen that oxidation occurs at 1150 °C for samples B1, B3, and B4, while the mass gain starts at 1050 °C for sample B2, below which Ca-α-SiAlON is stable in air atmosphere. It is also noted that the percentage of mass gain at 1400 °C varies: 3.2%, 3.5%, 2.2%, and 2.5% for samples B1, B2, B3, and B4, respectively. The smaller percentage mass gain value indicated a better oxidation resistance of Ca-α-SiAlON. Generally speaking, the particle size has a significant effect on the oxidation of materials, and a smaller particle size may cause worse oxidation resistance [25]. Considering that the powder size distribution is exactly the same for samples B3 and B4 (Figure 3), the resistance of oxidation of sample B3 is better than that of sample B4. Comparing the oxidation behavior of samples B1, B2, B3, and B4, it can be seen that the resistance to oxidation of samples B1 and B2 is less than that of samples B3 and B4. This behavior can, in fact, be explained by many factors, which will be discussed in the following sections. According to the non-isothermal experimental results, a series of oxidation temperatures were chosen to carry out the isothermal experiments.

**Figure 6:**
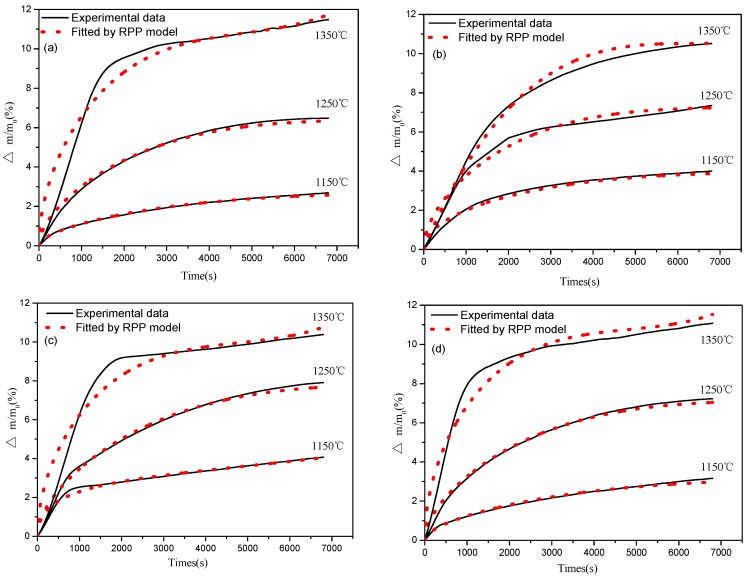
Isothermal oxidation curves for (**a**) sample B1; (**b**) sample B2; (**c**) sample B and (**d**) sample B4.

**Figure 7:**
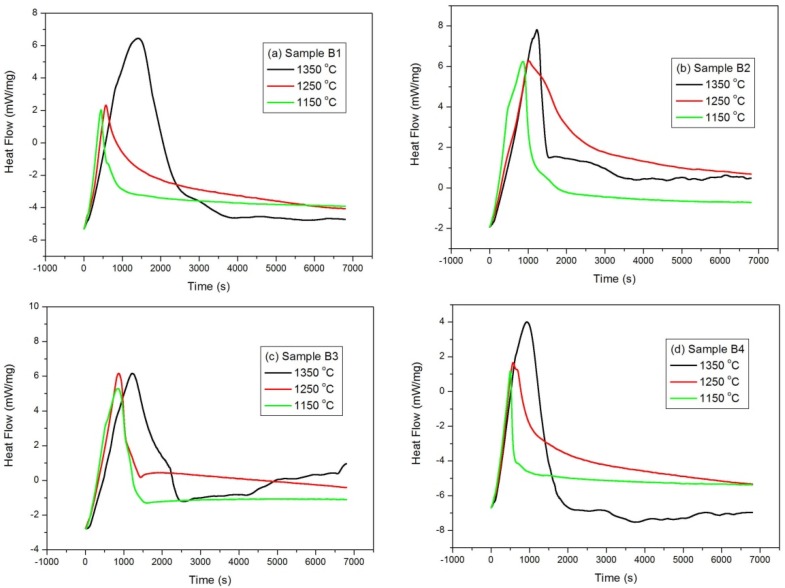
Differential Scanning Calorimetric (DSC) curves for (**a**) sample B1; (**b**) sample B2; (**c**) sample B3 and (**d**) sample B4, respectively.

Isothermal mass gain curves as a function of time for a series of oxidation temperatures in the range of 1150–1350 °C are shown in Figure 6, and it can be seen from the figures that all samples showed similar oxidation behavior, that is, a parabolic curve was observed. The oxidation rate shows an increase at the initial stage and a decrease at longer time intervals. This indicates that diffusion may be the controlling step due to the formation of a protective oxidation layer formed at the surface of the studied samples. It is also worth note that the relative mass gains are also variable. The thermal behaviors of different samples were also examined, and the differential scanning calorimetric (DSC) curves are illustrated in Figure 7. A sharp exothermal peak was observed for samples at different isothermal temperatures. It is also worth note that the exothermal peak at the initial stage is very sharp, suggesting that the oxidation rate is very drastic, initially. As the oxidation time proceeded for around 2500 s, the DSC curves became very mild, indicating that the oxidation rate is slow at longer time intervals.

### 2.3. Characterization of Oxidation Products

**Figure 8:**
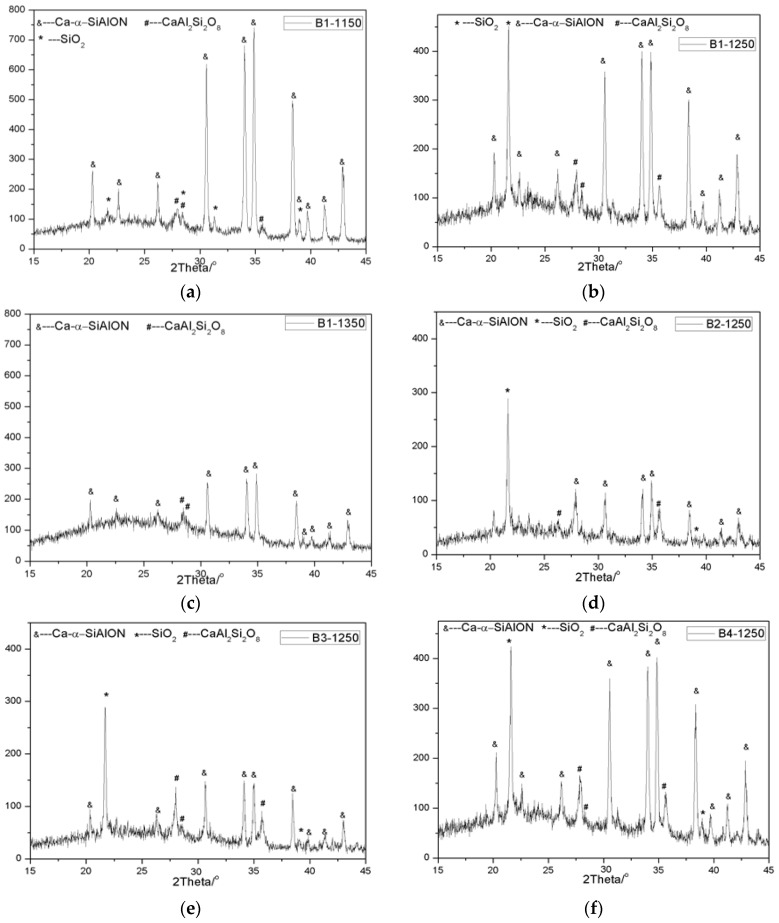
XRD patterns of the samples oxidized under different conditions: (**a**) B1-1150; (**b**) B1-1250; (**c**) B1-1350; (**d**) B2-1250; (**e**) B3-1250; (**f**) B4-1250.

**Figure 9:**
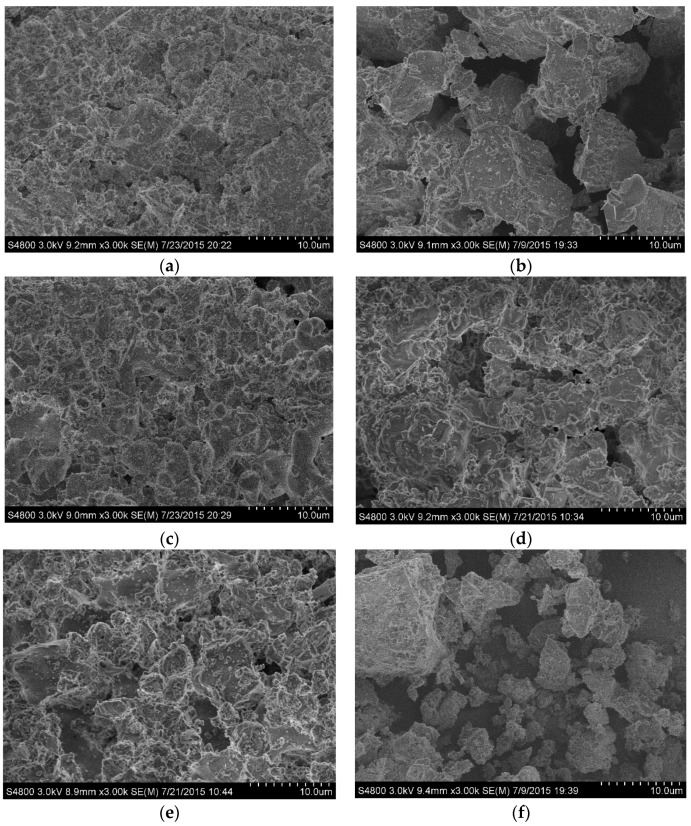
SEM images of the samples after 2 h oxidation under different conditions: (**a**) B1-1150; (**b**) B1-1250; (**c**) B1-1350; (**d**) B2-1250; (**e**) B3-1250; (**f**) B4-1250.

In order to determine the product of oxidation, the samples after oxidation at different temperatures were taken out and examined by X-ray diffraction (XRD) and scanning electron microscopy (SEM). The results are shown in Figure 8 and Figure 9, respectively. It can be seen that the oxidation products are dependent on oxidation temperature and sample, according to XRD patterns. At lower oxidation temperatures, such as 1150 °C, after oxidation for 2 h, XRD results showed that the oxidation products of Ca-α-SiAlON were composed of SiO_2_ and CaAl_2_Si_2_O_8_. Upon increasing oxidation temperature to 1250 °C, the relative intensity of SiO_2_ and CaAl_2_Si_2_O_8_ are obviously increased, and the relative intensity of Ca-α-SiAlON correspondingly decreased, suggesting that the oxidation rate accelerated. At a temperature of 1350 °C, it is interesting to note that the XRD peaks of SiO_2_ disappeared, instead of the appearance of an obvious boultin peak, suggesting that glassy phases were formed. This is consistent with the previous results showing that a glassy phase with a low melting point is easy to form in the SiO_2_-Al_2_O_3_-CaO ternary system [20,21,26]. The glassy phase with a low melting point may result in different oxidation behavior at high oxidation temperatures. After oxidation, the oxidized powders were taken out and examined by SEM. Figure 9 shows the morphological development of Ca-α-SiAlON powders under different conditions. It can be seen, compared with the raw materials, that the rod-shape structure of Ca-α-SiAlON was completely destroyed and the sample surface became very rough.

### 2.4. Kinetic Models

Generally speaking, the oxidation of Ca-α-SiAlON is a complicated process. The complete oxidation products are composed of CaO, SiO_2_, and Al_2_O_3_, and these phases can react with each other to form gehlenite (CaAl_2_Si_2_O_8_)—the reaction can be expressed as follows.

(1) $$Ca_{0.68}Si_{9.96}Al_{2.04}O_{0.68}N_{15.32}+O_{2}\to SiO_{2}+CaAl_{2}Si_{2}O_{8}+N_{2}$$

As the oxidation temperature increases, CaO, SiO_2_ and Al_2_O_3_ oxides may form a glassy phase at the eutectic point in the ternary CaO-SiO_2_-Al_2_O_3_ phase diagram [26], resulting in the disappearance of SiO_2_ in the oxidation products.

As in the aforementioned analysis (Figure 6), the oxidation of Ca-α-SiAlON powders is a typical gas-solid reaction. The thermogravimetric (TG) curves show a parabolic shape, indicating that the oxidation process may be controlled by mixed chemical reaction and diffusion. Hence, beyond the initial period, the oxidation reaction can be considered to consist of the following steps [24]:

(1) Oxygen transfer from bulk gas flow through the gas–solid boundary layer to the sample surface,
(2) Oxygen diffusion from the surface to the interface through the product layer,
(3) Chemical reactions between the sample and oxygen at the interface,
(4) Nitrogen diffusion through the interface to the sample surface,
(5) Nitrogen transfer from the sample surface to bulk gas flow through the gas-solid boundary layer.

Based on the explicit derivation of gas-solid reactions according to these steps, Chou *et al.* [14–18] have proposed a model (RPP model) that can deal with the gas-solid reaction under the conditions where all kinds of possible controlling steps exist, based on physical principles and mathematical calculation. The present study attempted to use this model to investigate the oxidation behavior of Ca-α-SiAlON powders synthesized under different conditions. Therefore, the reacted fraction ξ, for the case of describing the chemical reactions as the controlling step can be expressed as

(2) $$\xi =1-{\left(1-\frac{K_{O}(\sqrt{P_{O_{2}}}-\sqrt{P_{O_{2}}^{eq}})}{R_{0}v_{m}}\exp\left(\frac{-\Delta E_{c}}{RT}\right)t\right)}^{3}$$

where $\Delta E_{1}$ and ξ represent the apparent activation energy describing the chemical reaction and the reacted fraction, respectively. $P_{O_{2}}$ and $P_{O_{2}}^{eq}$ are the oxygen partial pressure in the bulk gas phase and the oxygen partial pressure in equilibrium at the reaction interface, respectively. $K_{O}$ is the temperature-independent constant. $v_{m}$ is the density of the sample, *R*_0_ is the initial radius of the particle, and *t* is the reaction time. *R* and *T* are the gas constant and temperature, respectively.

In the case of diffusion controlling the oxidation process, the reacted fraction ξ can be expressed as a function of time, temperature, oxygen partial pressure, and particle size. The relation can be described as

(3) $$\xi =1-{\left(1-\sqrt{\frac{2K_{O}^{0\beta }D_{O}^{0\beta }(\sqrt{P_{O_{2}}}-\sqrt{P_{O_{2}}^{eq}})}{R_{0}^{2}v_{m}}\exp\left(\frac{-\Delta E_{d}}{RT}\right)t}\right)}^{3}$$

where $\Delta E_{2}$ is the apparent activation energy describing diffusion. $K_{O}^{0\beta }$ and $D_{O}^{0\beta }$ are the constants independent of temperature but related to the materials investigated.

In order to simplify Equations (2) and (3), a new concept of the characteristic reaction time for the chemical and diffusion controlling steps can be introduced and expressed by the following equations, respectively:

(4) $$t_{c_{c}}=\frac{1}{\frac{K_{O}(\sqrt{P_{O_{2}}}-\sqrt{P_{O_{2}}^{eq}})}{R_{0}v_{m}}\exp\left(\frac{-\Delta E_{c}}{RT}\right)}$$

(5) $$t_{c_{d}}=\frac{1}{\frac{2K_{O}^{0\beta }D_{O}^{0\beta }(\sqrt{P_{O_{2}}}-\sqrt{P_{O_{2}}^{eq}})}{R_{0}^{2}v_{m}}\exp\left(\frac{-\Delta E_{d}}{RT}\right)}$$

According to Equations (4) and (5), it can be seen that the “characteristic reaction time” $t_{c_{c}}$ and $t_{c_{d}}$ are dependent on such factors as the radius of the powder, the reaction intensity of the environment, the character of the medium where gas passes through, as well as the property of reactant and product materials. It has no relation with the oxidation time, however. Therefore, the characteristic reaction time can be selected to characterize the property of oxidation resistance of Ca-α-SiAlON powders. In general, the “characteristic reaction time” can also be extracted from a set of experimental data. Substituting Equations (4) and (5) into Equations (2) and (3), respectively, yields:

(6) $$\xi =1-{\left(1-\frac{t}{t_{c_{c}}}\right)}^{3}$$

(7) $$\xi =1-{\left(1-\sqrt{\frac{t}{t_{c_{d}}}}\right)}^{3}$$

When $t=t_{c}$, ξ = 1. That is, $t_{c}$ is the time required for the whole powder to undergo complete oxidation. $\Delta E_{c}$ and $\Delta E_{d}$ can also be calculated from the value of $t_{c_{c}}$ and $t_{c_{d}}$, according to the following equations:

(8) $$\ln t_{c_{c}}=\frac{\Delta E_{c}}{RT}-\ln B_{c}$$

(9) $$B_{c}=\frac{K_{O}(\sqrt{P_{O_{2}}}-\sqrt{P_{O_{2}}^{eq}})}{R_{0}v_{m}}$$

(10) $$\ln t_{c_{d}}=\frac{\Delta E_{d}}{RT}-\ln B_{d}$$

(11) $$B_{d}=\frac{R_{0}^{2}v_{m}}{2K_{O}^{0\beta }D_{O}^{0\beta }(\sqrt{P_{O_{2}}}-\sqrt{P_{O_{2}}^{eq}})}$$

Based on the above illustration, the isothermal oxidation kinetics of Ca-α-SiAlON is given as:

(12) $$\xi =\left(1-{\left(1-\frac{t}{t_{c_{c}}}\right)}^{3}\right)+\left(1-{\left(1-\sqrt{\frac{t}{t_{c_{d}}}}\right)}^{3}\right)$$

Note that the y-axis in the experimental plots (Figure 6) is the mass gain percent ∆*w*(∆*m*/*m*_0_) instead of the reacted fraction ξ. A transformation is required according to the following equation:

(13) $$\xi =\frac{\Delta m/m_{0}}{\Delta m_{\max}/m_{0}}$$

Where $\Delta m_{max}$ is the theoretical maximum increment after complete oxidation. By the regression method, characteristic oxidation time ($t_{c_{c}}$ and $t_{c_{d}}$) and apparent activation energy of $\Delta E_{c}$ and $\Delta E_{d}$ under the three temperatures are summarized and illustrated in Table 1. The curves obtained from the above equations are also shown in Figure 6 as red lines, which fits the experimental data fairly well.

**Table 1:** The characteristic time and apparent activation energy of sample oxidation calculated by RPP model in air at 1150–1350 °C.

Sample	Temperature (°C)	Characteristic Time (s)		Apparent Activation Energy (kJ/mol)	
		*t*c_c_	*t*c_d_	$\Delta E_{c}$	$\Delta E_{d}$
B1	1150	9846	18,050	80.95	51.98
	1250	7409	16,055		
	1350	4206	10,429		
B2	1150	8586	15,790	28.94	49.25
	1250	7090	14,055		
	1350	6360	9392		
B3	1150	13,197	23,619	85.83	56.99
	1250	8548	17,046		
	1350	4378	12,820		
B4	1150	10,603	20,488	105.29	58.70
	1250	7918	16,893		
	1350	4301	11,259		

Theoretically, when analyzing Table 1, it can be seen that the value of characteristic oxidation time gradually decreases with increasing temperature for each sample, indicating the oxidation resistance of the sample decreases with increasing temperature. On the other hand, the characteristic oxidation time increases in the sequence of samples B2, B1, B4, and B3 at the same temperature. Theoretically speaking, a shorter characteristic oxidation time usually indicates inferior oxidation resistance properties and the results agree well with that as shown in Figure 5. It should be also noted, however, that particle size is also closely related to its oxidation resistance property, and a decreasing particle size is thought to be detrimental to material oxidation resistance [25]. According to the particle size distribution (Figure 3), the particle size of samples B1 and B2 is larger than that of samples B3 and B4. If the theory fits in our experiment, sample B1 should have the longest characteristic oxidation time, followed by sample B2. In addition, the characteristic oxidation time for samples B3 and B4 should be similar to each other and shorter than that of sample B2. This speculation contradicts with our experimental results, however—the other factors may also have a significant effect on its oxidation resistance properties. Based on the SEM images of Ca-α-SiAlON (Figure 2), it is concluded that crystal morphology may also determine oxidation resistance. From Figure 2, it is found that hexagonal column-shaped crystals exist in all samples apart from sample B2, which has the worst oxidation resistance, validating the theory that hexagonal column-shaped crystals are beneficial to enhanced material oxidation resistance.

Based on these findings, it can be concluded that the addition of additives could cause a change in crystal morphology and further affect oxidation properties. To be more specific, the addition of 5 wt.% NH_4_F leads to the formation of irregular grain-shaped Ca-α-SiAlON crystals with a smaller particle size than sample B1. These two factors result in the worst oxidation resistance property for sample B2, confirming that NH_4_F addition has a negative effect on material oxidation resistance. When compared to the other three samples, it is found that sample B1 has the least amount of hexagonal column-shaped crystals. As a result, samples B3 and B4 have better oxidation resistance than sample B1. Regarding samples B3 and B4, the addition of 5% NH_4_Cl in B3 could better promote the formation of hexagonal column-shaped Ca-α-SiAlON crystals than the addition of mixed additives (2.5 wt.% NH_4_F and 2.5 wt.% NH_4_Cl). Therefore, the oxidation resistance of sample B3 is better than that of B4.

## 3. Experimental Section

### 3.1. Sample Preparation

Ca-α-SiAlON powders studied in this paper were fabricated by combustion synthesis in a pressurized N_2_ atmosphere. Raw materials included Si, Al, Si_3_N_4_, NH_4_F, NH_4_Cl, and CaO. Nitrides and NH_4_F or NH_4_Cl are used as additives to reduce the agglomeration of metal droplets and facilitate the infiltration of N_2_. The raw materials are mixed according to the general formula of Ca*_x_*Si_12__−(_*_m_*_+_*_n_*_)_Al*_m_*_+_*_n_*O*_n_*N_16__−_*_n_*, where Ca^2+^ was used as the stabilizing metallic cation because of its cost and availability for large-scale industrial production compared to rare-earth elements. The composition with *m* = 2 and *n* = 1 was investigated for the synthesis of Ca-α-SiAlON in the present study. The designated starting compositions of all samples are listed in Table 2. The starting powder mixtures were prepared by using CaO, NH_4_F, NH_4_Cl, α-Si_3_N_4_ (A.R., Beijing Chemical Co., Beijing, China), Al, and Si (99.5%, Fushun Al Factory, Fushun, China).

**Table 2:** Starting compositions of the samples in combustion synthesis of Ca-α-SiAlON.

Sample	CaO/mol	Si/mol	Si_3_N_4_/mol	Al/mol	NH_4_F/wt.%	NH_4_Cl/wt.%
B1	1	3.6	1.8	2.5	0	–
B2	1	3.6	1.8	2.5	5%	–
B3	1	3.6	1.8	2.5	–	5%
B4	1	3.6	1.8	2.5	2.5%	2.5%

The carefully weighted raw materials were mixed by agate balls in a plastic jar for 24 h with absolute ethanol used as medium. The obtained slurry was dried in an oven at 70 °C for 8 h and then sieved. The reactant powder mixture was pressed into a pellet to increase intimate contact between these materials. The products of reaction are extremely porous, around 50% of the theoretical density. Then, the reactant mixture is placed in a graphite crucible, and placed into a combustion chamber. The chamber is evacuated and then filled with high-purity N_2_. The combustion reaction was triggered by passing an electric current through a tungsten coil close above the sample. It should be pointed out that once the combustion synthesis reaction is triggered, the combustion temperature immediately increases from room temperature to nearly 1800 °C in one second, and the pressure in the system correspondingly dramatically increases to the maximum value of 20 MPa. When the combustion reaction is over, the sample quickly cools down at a cooling rate of ~60 °C/s. The Ca-α-SiAlON products are thus obtained.

### 3.2. Apparatus and Procedure

The oxidation of Ca-α-SiAlON powders were carried out on a SETARAM Instrument (Setsys Evolution, S60/58341, SETARAM Instrumentation, France) with a detection limit of 1 µg, which was fully controlled by a controller. Before the experiment, four standard samples (Al, Ag, Au, and Ni) were first used to calibrate the instrument. The oxidation samples were carefully weighed and put in a suitable platinum container in the transducers. In order to overcome the buoyancy effect during measurement, a series of experiments were repeated in a platinum crucible with the same amount of a-Al_2_O_3_ powders as a reference.

In order to choose the oxidation temperature range, a series of non-isothermal experiments were carried out. The carefully weighted Ca-α-SiAlON powders were placed in the furnace and the air was let into the reaction tube at a fixed flow rate of 80 mL/min. The furnace was then heated from room temperature to a maximum temperature of 1400 °C at fixed heating rates of 20 °C/min. After the non-isothermal experiments, a series of temperatures were chosen to carry out the isothermal oxidations. The TG setup was first evacuated for 5 min, then flushed with high purity argon gas, then the furnace was rapidly heated to the required temperature under flowing purified argon gas. After maintaining the required temperature for 5 min to establish the thermal equilibrium, air was let into the reaction tube instead of the argon atmosphere at a flow rate of 80 mL/min. A series of preliminary experiments were carried out at different flow rates to ensure that this gas flow was above the starvation rate. At the end of the experimental run, the reaction was stopped by using high purity argon gas. The powders after oxidation were taken and analyzed by Scanning Electronic Microscopy (SEM). Then, the oxidized powders were measured using the X-ray Diffraction (XRD) patterns in a 2θ range of 10° to 70° using Cu K_α_ radiation of 40 kV and 30 mA with a scanning rate of 4° min^−1^.

## 4. Conclusions

Ca-α-SiAlON powders were synthesized with different additives by combustion synthesis (CS) and further examined thermogravimetrically (TG) to analyze its oxidation resistance properties. The oxidation products were confirmed to be composed of SiO_2_ and CaAl_2_Si_2_O_8_ at low oxidation temperatures, whereas the SiO_2_-Al_2_O_3_-CaO ternary glassy phase was formed at higher temperatures. Based on the TG results, it is suggested that oxidation was controlled by mixed chemical and diffusion steps and was successfully fitted by Chou’s model. Kinetic oxidation indicated that the addition of NH_4_F in sample B2 caused the formation of a large amount of grain-shaped Ca-α-SiAlON crystals with a smaller particle size than sample B1, which led to the worst oxidation property. In contrast, the addition of NH_4_Cl in sample B3, and mixed additives composed of NH_4_F and NH_4_Cl in sample B4, promoted the formation of hexagonal column-shaped Ca-α-SiAlON crystals, resulting in better oxidation resistance. The oxidation resistance for these three samples from best to the worst was in the sequence: B3, B4, B1.
